# Self-Reported Cognitive Function and Mental Health Diagnoses among Former Professional American-Style Football Players

**DOI:** 10.1089/neu.2019.6661

**Published:** 2020-04-09

**Authors:** Franziska Plessow, Alvaro Pascual-Leone, Caitlin M. McCracken, Jillian Baker, Supriya Krishnan, Aaron Baggish, Ann Connor, Theodore K. Courtney, Lee M. Nadler, Frank E. Speizer, Herman A. Taylor, Marc G. Weisskopf, Ross D. Zafonte, William P. Meehan

**Affiliations:** ^1^ Football Players' Health Study at Harvard University, Harvard Medical School, Boston, Massachusetts.; ^2^ Neuroendocrine Unit, Department of Medicine, Massachusetts General Hospital and Harvard Medical School, Boston, Massachusetts.; ^3^ Hinda and Arthur Marcus Institute for Aging Research and Center for Memory Health, Department of Neurology, Hebrew SeniorLife and Harvard Medical School, Boston, Massachusetts.; ^4^ Guttmann Brain Health Institut, Institut Guttmann, Universitat Autònoma de Barcelona, Barcelona, Spain.; ^5^ College of Pharmacy, Oregon State University/Oregon Health Science University, Portland, Oregon.; ^6^ Cardiovascular Performance Program, Department of Medicine, Massachusetts General Hospital and Harvard Medical School, Boston, Massachusetts.; ^7^ Berenson-Allen Center for Noninvasive Brain Stimulation and Division for Cognitive Neurology, Department of Neurology, Beth Israel Deaconess Medical Center and Harvard Medical School, Boston, Massachusetts.; ^8^ Department of Environmental Health, Harvard T. H. Chan School of Public Health, Boston, Massachusetts.; ^9^ Department of Medicine, Dana-Farber Cancer Institute, Boston, Massachusetts.; ^10^ Channing Division of Network Medicine, Department of Medicine, Brigham and Women's Hospital and Harvard Medical School, Boston, Massachusetts.; ^11^ Department of Medicine, Cardiovascular Research Institute, Morehouse Medical School, Atlanta, Georgia.; ^12^ Department of Physical Medicine and Rehabilitation, Brigham and Women's Hospital, Massachusetts General Hospital, Spaulding Rehabilitation Hospital, and Harvard Medical School, Boston, Massachusetts.; ^13^ Department of Pediatrics and Orthopedics, Boston Children's Hospital and Harvard Medical School, Boston, Massachusetts.

**Keywords:** cognitive function, football, mental health, quality of life

## Abstract

Clinical practice strongly relies on patients' self-report. Former professional American-style football players are hesitant to seek help for mental health problems, but may be more willing to report cognitive symptoms. We sought to assess the association between cognitive symptoms and diagnosed mental health problems and quality of life among a cohort of former professional players. In a cross-sectional design, we assessed self-reported cognitive function using items from the Quality of Life in Neurological Disorders (Neuro-QOL) Item Bank. We then compared mental health diagnoses and quality of life, assessed by items from the Patient-Reported Outcome Measurement Information System (PROMIS^®^), between former professional players reporting daily problems in cognitive function and former players not reporting daily cognitive problems. Of the 3758 former professional players included in the analysis, 40.0% reported daily problems due to cognitive dysfunction. Former players who reported daily cognitive problems were more likely to also report depression (18.0% vs. 3.3%, odds ratio [OR] = 6.42, 95% confidence interval [CI] [4.90–8.40]) and anxiety (19.1% vs. 4.3%, OR = 5.29, 95% CI [4.14–6.75]) than those without daily cognitive problems. Further, former players reporting daily cognitive problems were more likely to report memory loss and attention deficit(/hyperactivity) disorder and poorer general mental health, lower quality of life, less satisfaction with social activities and relationships, and more emotional problems. These findings highlight the potential of an assessment of cognitive symptoms for identifying former players with mental health, social, and emotional problems.

## Introduction

Mental health and quality of life among professional American-style football (ASF) players beyond their active playing years has become a key area of interest in sports medicine. Evidence suggests that careers in professional football may be associated with long-term neurocognitive sequelae and mental health problems,^1–7^ and mental health problems may be more prevalent among active and/or former professional ASF players with a history of concussion.^3,8–12^ In a cross-sectional study of 2552 members of the National Football League (NFL) Players Association – Retired Section, Guskiewicz and colleagues reported a lifetime prevalence of depression of 11.1% and a positive association between the number of recalled concussions and diagnosis of depression.^8^ In a follow-up study of >1000 members of the original sample, Kerr and coworkers reported a 9 year incidence of depression of 10.2%, with the incidence increasing as the number of recalled concussions increased.^9^ In comparison, a nationwide survey determined the percentage of men with current depression in the general United States population to be 5.5% for 20–39-year olds, 5.2% for 40–59-year olds, and 6.1% among ≥60 years of age.^13^

The Football Players' Health Study at Harvard University is designed to pursue research that can lead to improvements in health and quality of life among former professional ASF players.^14,15^ A core element of our research approach is the close collaboration among scientists, former professional ASF players, and their families when identifying, prioritizing, and pursuing medical problems of interest. Early in the course of the study, our focus groups revealed that, whereas former professional players are willing to report cognitive difficulties, which are viewed by many as related to repeated impacts to the head during play, they are reluctant to report mental health problems, such as depression and anxiety. A recent qualitative study interviewing 25 current and former NFL players together with 27 family members revealed in 15 interviews that concerns around managing mental health challenges created a barrier to seeking support for experienced mental distress.^16^

These observations are further supported by the literature. Even though the culture of football may be growing more accepting of self-reported physical injuries during play,^17^ the self-report of emotional problems does not seem to have evolved at the same rate. Men, in general, are less likely to report or seek treatment for mental health problems.^18–20^ Former professional ASF players may be even less inclined to report or seek help for non-physical injuries.^21^ This is especially problematic, as clinical practice strongly relies on the self-reporting of mental health problems. Moreover, this bias is of immediate clinical relevance, as many former professional players may be experiencing symptoms of depression and anxiety that are not reported to their treating clinicians, particularly considering the high prevalence rates of mood disorders in middle-aged men.^13^

Because our focus groups reported a willingness to acknowledge cognitive symptoms, we sought to determine whether the reporting of cognitive symptoms was associated with a diagnosis of depression and/or anxiety. If so, the predictive value of reported cognitive function as related to potentially treatable mental health problems might prove useful to clinicians caring for former professional players as a means of screening for mental health problems. To this end, we analyzed data from an initial intake questionnaire of a cohort study of former professional ASF players and tested the following hypotheses: Former professional players who report daily cognitive problems are more likely to have (1) a diagnosis of depression and/or anxiety by a health professional leading to prescribed pharmacological treatment, (2) a lower quality of life, and (3) unhealthy lifestyle habits (frequent alcohol consumption, cigarette smoking, and/or low levels of exercise).

## Methods

### Study sample

In February 2015, the Football Players' Health Study at Harvard University identified 14,538 former active professional players who had signed a contract with the NFL between 1960 and 2013 and were no longer listed as active players and believed to be alive and eligible. This start year 1960 was chosen, as the transition to the helmet with a hard plastic shell, which was accompanied by major rule changes, was complete and well established by 1960. Of these 14,538 players, we had potential contact information for 13,403 former professional players, all of whom we attempted to contact. We believe that we reached 13,200 potential study participants. At the time of this analysis, 3779 former professional ASF players (28.6%) had responded. Newly retired players continue to enroll, and we plan to follow them longitudinally. The four-page questionnaire assessing self-reported sociodemographic, play-related, health, quality of life, and lifestyle characteristics was sent in either paper or electronic format. In our sample, 1308 former players completed the questionnaire in paper format and 2471 completed it via Research Electronic Data Capture (REDCap^TM^). This study was approved by the Institutional Review Board (IRB) of Beth Israel Deaconess Medical Center, an affiliate of Harvard Medical School. The IRBs at the collaborating Harvard institutions Harvard Medical School, Boston Children's Hospital, and Massachusetts General Hospital ceded IRB review to the Beth Israel Deaconess Medical Center's IRB's review. Consent was obtained via an implied consent model with the participant's willingness to complete the questionnaire serving as implied informed consent. For additional details regarding the design and objectives of the Football Players' Health Study, study sample, and determination of eligibility, please refer to the study by Zafonte and coworkers.^22^

### Cognitive function

We assessed cognitive function using items from the Quality of Life in Neurological Disorders (Neuro-QOL) Item Bank v1.0 for Applied Cognition – General Concerns,^23^ an established reliable and valid tool for assessing perceived difficulties in memory, attention, and decision making. Each item describes a cognitive problem (e.g., “I had trouble thinking clearly”) and asks respondents to indicate how frequently they have experienced this problem over the past 7 days. Answers are provided on a five-point Likert-like scale including the options “never,” “rarely (once),” “sometimes (two or three times),” “often (about once a day),” and “very often (several times a day).” The item bank provides a total of 18 questions loading on a single latent construct (applied cognition) and allows for selection of items of highest relevance to a target population with the recommendation to use at least eight items in order to reliably estimate the ability reflected by the latent construct.^23^ The 11 items used in this study (Table 1) were pilot tested on former professional ASF players prior to inclusion in the initial intake questionnaire.

**Table 1.** Frequency (%) of Self-Reported Concerns Regarding Cognitive Function in 3758 Former Professional American-Style Football Players Captured with 11 Questions from the Quality of Life in Neurological Disorders (Neuro-QOL) Item Bank v1.0 for Applied Cognition: General Concerns^a^

Item	Never	Rarely (once)	Sometimes (2–3 times)	Often (about once a day)	Very often (several times a day)
In the past 7 days,					
1. I had to read something several times to understand it.	806 (21.5)	1092 (29.1)	1,057 (28.1)	405 (10.8)	397 (10.6)
2. I had trouble keeping track of what I was doing if I was interrupted.	873 (23.2)	975 (26.0)	1,022 (27.2)	475 (12.6)	412 (11.0)
3. I had difficulty doing more than one thing at a time.	1036 (27.7)	996 (26.6)	891 (23.8)	432 (11.5)	392 (10.5)
4. I had trouble remembering new information, like phone numbers or simple instructions.	894 (23.8)	944 (25.1)	916 (24.4)	509 (13.6)	494 (13.2)
5. I had trouble thinking clearly.	1144 (30.5)	1054 (28.1)	830 (22.1)	412 (11.0)	316 (8.4)
6. My thinking was slow.	1142 (30.6)	1031 (27.6)	832 (22.3)	434 (11.6)	296 (7.9)
7. I had to work really hard to pay attention or I would make a mistake.	1197 (32.0)	999 (26.7)	782 (20.9)	423 (11.3)	346 (9.2)
8. I had trouble concentrating.	987 (26.4)	986 (26.4)	905 (24.2)	453 (12.1)	409 (10.9)
9. I had trouble remembering whether I did things I was supposed to do.	953 (25.4)	991 (26.4)	924 (24.7)	492 (13.1)	388 (10.4)
10. I had trouble making decisions.	1334 (35.7)	1046 (28.0)	762 (20.4)	328 (8.8)	266 (7.1)
11. I had trouble planning out steps of a task.	1443 (38.7)	1007 (27.0)	717 (19.2)	332 (8.9)	234 (6.3)

^a^ Twenty-one out of 3779 participants provided valid responses for <8 out of 11 Neuro-QOL items and were not included in the analysis. Total values of <3758 for a question indicate missing responses.

### Mental health

Respondents were defined as having depression, anxiety, memory loss, or attention deficit(/hyperactivity) disorder (ADD/ADHD), if they reported currently taking medication prescribed by a healthcare professional for the treatment of that disorder. We chose this conservative definition, as there are common misconceptions regarding mental health diagnoses among the general population.^24^ In addition, we assessed the correlation between cognitive symptoms and self-reported current symptoms of depression and/or anxiety using sum scores on the Neuro-QOL and the Patient Health Questionnaire for Depression and Anxiety (PHQ-4). The four-item PHQ-4 represents a screening tool for depression and anxiety in outpatient or home settings. Based on self-reported symptom presence over the past 2 weeks on a four-point Likert-like scale from 0 (“not at all”) to 3 (“nearly every day”), the sum score distinguishes between no (0–2), mild (3–5), moderate (6–8), and severe (9–12) depression and/or anxiety, providing a quantitative measure of current symptom severity in addition to the more conservative definition of medical management.^25^ Finally, we used the established cutoff of a PHQ-4 score of ≥6 for a positive screening result for current depressive and/or anxiety symptoms (moderate to severe symptoms).^26^

### Quality of life

Quality of life was assessed employing the widely used Global Health Scale 10-Item Bank v1.0/1.1 of the Patient-Reported Outcome Measurement Information System (PROMIS^®^).^27^ The items for general quality of life (Global02), mental health (Global04), satisfaction with social activities and relationships (Global05), and emotional problems (Global10) were answered on a five-point Likert-like scale with the categories “poor,” “fair,” “good,” “very good,” and “excellent.”

### Other measures

Alcohol use was measured by the number of alcoholic beverages (i.e., 12 oz. of beer, 5 oz. of wine, or one shot [2 cL] of alcohol, such as gin, rum, or vodka) consumed weekly. The measure was created based on two questions that asked about (1) the number of days a participant drank alcoholic beverages during a typical week and (2) the number of drinks consumed on a typical day that the person drank. Frequent drinking was defined as consuming ≥15 alcoholic beverages weekly, which is the Centers for Disease Control and Prevention's classification for heavy drinking.^28^

Age, height, weight, race (American Indian/Alaskan Native, Asian, black/African American, Native Hawaiian/Pacific Islander, white, and other), ethnicity (Hispanic/Latino: yes/no), currently employed (yes, in football, yes, outside of football, retired, and no), domestic status (living with partner: yes/no), seasons of professional football played, position played (positions mirroring each other on the offense and defense were grouped together as appropriate, taking into account the type and number of physical impacts and body composition of the player: offensive/defensive lineman, linebacker, defensive back/wide receiver, running back, tight end, quarterback, kicker/punter), current cigarette use (yes/no), and diagnosis of sleep apnea (yes/no) were self-reported. For race and position played, participants could mark all options that applied.

Exercise regimen was assessed by self-reported weekly engagement in the following types of exercise: walking for exercise, jogging, running, other aerobic (e.g., bicycling, stationary/elliptical machine/other), low-intensity exercise (e.g., yoga, Pilates, stretching), and weight training (e.g., lifting free weights, using weight machines). Low level of exercise was defined conservatively as engaging in <1 h of accumulated exercise activity weekly.

### Data analysis

Outlier values were corrected where possible using publicly available data (e.g., for years played in the NFL) or were set to missing. For all analyses concerning cognitive function, participants were included only if they provided valid responses to at least 8 out of the 11 Neuro-QOL items, resulting in a final sample of 3758 former professional players.

Based on their responses to the Neuro-QOL items, respondents were divided into two groups: (1) former professional players who reported at least one daily problem with their applied cognitive abilities and (2) former professional players who did not report such daily concerns, and group differences were assessed using the χ^2^ test. For mental health outcomes, odds ratios adjusted (aOR) for age and sleep apnea, variables known to affect cognitive function,^29,30^ were determined using logistical regression analyses. In addition, to test whether more severe cognitive symptoms are associated with more depressive and/or anxiety symptoms among those with a diagnosis of depression and/or anxiety, we conducted a nonparametric correlation analysis of Neuro-QOL and PHQ-4 sum scores in this subgroup. Finally, we examined the relevance of the presence of current depression and/or anxiety for the depression/anxiety and cognitive function link. Using χ^2^ tests, we compared how many former professional players with a diagnosis of depression and/or anxiety but no current moderate to severe symptoms of depression and/or anxiety (PHQ-4 < 6) reported daily cognitive concerns (%) with that number among (1) former professional players with a diagnosis of depression and/or anxiety and current depression and/or anxiety (PHQ-4 ≥ 6) and (2) former professional players without a diagnosis of depression and/or anxiety and no current moderate or severe depressive and/or anxiety symptoms (PHQ-4 < 6).

## Results

Respondents ranged from 24 to 89 years of age (mean age: 52.77 years); 1.2% were American Indian or Alaska Native, 0.3% were Asian, 37.5% were black/African American, 1.1% were Native Hawaiian or other Pacific Islanders, 60.1% were white, and 1.7% were “other.” A total of 1.4% identified as Hispanic, 97.8% as identified as non-Hispanic, and 0.8% chose not to respond. They played an average of seven seasons in the NFL. All positions were represented (Table 2).

**Table 2.** Basic Characteristics (*n* = 3779 Former Professional American-Style Football Players)^a^

Characteristic	Mean ± SD or* n *(%)
Sociodemographics	
Age (years)	52.77 ± 14.14
Race^b^	
American Indian/Alaska Native	44 (1.2)
Asian	10 (0.3)
Black/African American	1417 (37.5)
Native Hawaiian/Other Pacific Islander	41 (1.1)
White	2272 (60.1)
Other	63 (1.7)
Ethnicity	
Hispanic/Latino	51 (1.4)
Not Hispanic/Latino	3593 (97.8)
Chose not to respond	30 (0.8)
Currently employed	
Yes, in football	355 (9.5)
Yes, outside of football	2172 (58.3)
Retired	700 (18.8)
No	497 (13.4)
Domestic status: living with partner	
Yes	3061 (81.8)
No	683 (18.2)
Play-related characteristics	
Number of seasons played professionally	6.78 ± 3.84
Position played^b^	
Offensive/Defensive line	1369 (36.2)
Linebacker	631 (16.7)
Defensive back/Wide receiver	973 (25.7)
Running back	410 (10.9)
Tight end	313 (8.3)
Quarterback	182 (4.8)
Kicker/Punter	208 (5.5)
Physical health	
BMI	31.22 ± 4.96
Sleep apnea	
Yes	843 (22.8)
No	2853 (77.2)

^a^ Total values of <3,779 for a question indicate missing responses. ^b^Participants had the option to select more than one category.

BMI, body mass index; SD, standard deviation.

Of the 3758 former professional ASF players included in the analysis, the majority reported having had at least one cognitive problem in the preceding 7 days (Table 1). More than a third (*n* = 1502; 40.0%) reported experiencing at least one cognitive problem on a daily basis, whereas 2256 former professional players (60.0%) did not report any daily cognitive problems. Former professional players with daily problems in cognitive function were more likely to have a diagnosis of depression, anxiety, memory loss, or ADD/ADHD (Table 3). These effects remained significant after adjusting for age and sleep apnea: depression: aOR = 6.17, 95% confidence interval (CI) [4.67–8.15]; anxiety: aOR = 5.06, 95% CI [3.93–6.52]; memory loss: aOR = 21.13, 95% CI [11.57–38.58]; and ADD/ADHD: aOR = 4.73, 95% CI [3.22–6.95].

**Table 3.** Mental Health, Quality of Life, and Lifestyle Factors in Former Professional American-Style Football Players Reporting Daily Problems in Cognitive Function^a^ Compared with Those Who Do Not Report Daily Cognitive Problems

Characteristic	Daily cognitive problems (*n* = 1502)	No daily cognitive problems (*n* = 2256)	OR [95% CI]
Mental health^b^			
Depression			
Yes	259 (18.0)	73 (3.3)	6.42
No	1183 (82.0)	2140 (96.7)	[4.90–8.40]
Anxiety			
Yes	276 (19.1)	94 (4.3)	5.29
No	1171 (80.9)	2109 (95.7)	[4.14–6.75]
Memory loss			
Yes	137 (9.5)	12 (0.5)	19.14
No	1300 (90.5)	2179 (99.5)	[10.56–34.66]
ADD/ADHD			
Yes	118 (8.1)	37 (1.7)	5.16
No	1334 (91.9)	2157 (98.3)	[3.54–7.51]
Quality of life			
General quality of life			
Poor/fair	553 (37.5)	147 (6.6)	8.48
Good to excellent	923 (62.5)	2080 (93.4)	[6.96–10.33]
Mental health			
Poor/fair	935 (62.7)	246 (11.0)	13.63
Good to excellent	557 (37.3)	1997 (89.0)	[11.51–16.14]
Satisfaction with social activities and relationships			
Poor/fair	725 (48.6)	244 (10.9)	7.73
Good to excellent	767 (51.4)	1995 (89.1)	[6.54–9.14]
Emotional problems			
Often/always	717 (47.9)	163 (7.3)	11.77
Never to sometimes	779 (52.1)	2085 (92.7)	[9.75–14.22]
Lifestyle			
Frequent drinking^c^			
Yes	326 (22.0)	447 (20.0)	1.13
No	1154 (78.0)	1787 (80.0)	[0.96–1.33]
Current smoking			
Yes	66 (4.4)	52 (2.3)	1.96
No	1419 (95.6)	2189 (97.7)	[1.35–2.83]
Low level of exercise^d^			
Yes	416 (27.7)	310 (13.8)	2.41
No	1084 (72.3)	1943 (86.2)	[2.04–2.84]

Data are presented as *n* (%).

^a^ Assessed with 11 questions from the Quality of Life in Neurological Disorders (Neuro-QOL) Item Bank v1.0 for Applied Cognition – General Concerns. Twenty-one out of 3779 participants provided valid responses for <8 out of 11 Neuro-QOL items and were not included in the analysis. Total values of <3758 for an outcome indicate missing responses. ^b^Based on self-report of currently taking medication for the treatment of that disorder. ^c^Defined as ≥15 alcoholic beverages (i.e., 12 oz. of beer, 5 oz. of wine, or one shot [2 cL] of alcohol, such as gin, rum, or vodka) per week based on the Centers for Disease Control and Prevention's classification for heavy drinking.^26^

^d^ Defined as <1 h/week of walking, jogging, running, other aerobic (e.g., bicycling, stationary/elliptical machine/other), low-intensity exercise (e.g., yoga, Pilates, stretching), and/or weight training.

ADD, attention deficit disorder; ADHD, attention deficit/hyperactivity disorder; CI, confidence interval; OR, odds ratio.

**FIG. 1.**
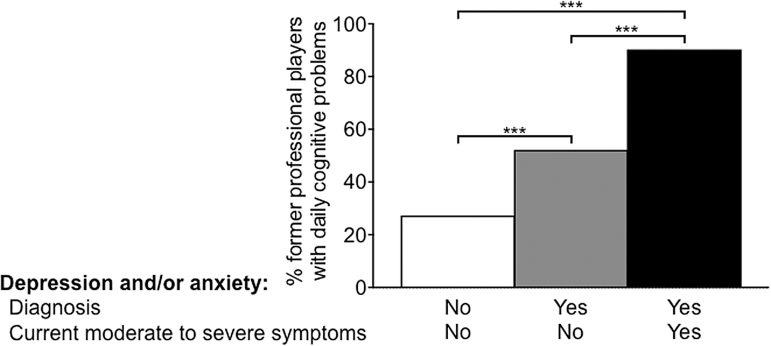
Percentage of former professional American-style football players reporting daily cognitive problems depending on (1) diagnosis of depression and/or anxiety (defined by currently prescribed medication for these conditions by a healthcare professional; yes/no) and (2) current experience of moderate to severe depressive and/or anxiety symptoms (defined by a Patient Health Questionnaire for Depression and Anxiety [PHQ-4] score ≥6; yes/no). ***p < 0.001.

Among former professional ASF players diagnosed with depression and/or anxiety who also completed the PHQ-4, we found a linear relationship between Neuro-QOL and PHQ-4 sum scores, indicating that in this subgroup, worse cognitive function was associated with more pronounced depressive and/or anxiety symptoms, *r_s_* = 0.65, *p* < 0.0001. Also, among former professional players with a diagnosis of depression and/or anxiety who did not report significant current depressive and/or anxiety symptoms, 52.2% reported daily cognitive concerns. This percentage was smaller than the percentage of former professional ASF players with a diagnosis of depression and/or anxiety who also reported current depression and/or anxiety (90.3%; *p* < 0.001), but larger than the proportion of former professional players who were not diagnosed with depression and/or anxiety and did not report current moderate to severe depressive and/or anxiety symptoms (27.3%; *p* < 0.001). In addition, former professional players with a diagnosis and significant current depressive and/or anxiety symptoms differed from those with neither the diagnosis of anxiety and/or depression nor current symptoms (*p* < 0.001; Fig. 1).

Compared with those without daily cognitive problems, former professional ASF players with problems in memory, attention, and/or decision making on a daily basis also reported a lower general quality of life, poorer mental health, less satisfaction with social activities and relationships, and more emotional problems (Table 3). Finally, former professional players experiencing daily problems in their cognitive function were more likely to be smokers and to report exercising <1 h per week. No difference in the report of alcohol consumption was found between groups (Table 3).

## Discussion

Former professional ASF players who experienced daily cognitive problems had higher odds of also carrying the diagnosis of at least one of the following: depression, anxiety, memory loss, or ADD/ADHD than those who did not report day-to-day cognitive issues (Hypothesis 1). An additional analysis in the subgroup of former professional players diagnosed with depression and/or anxiety showed a linear correlation between the extent of cognitive problems and the severity of depressive and/or anxiety symptoms, suggestive of an association between cognitive and emotional problems in this population. Further, we observed that among those former players with a diagnosis of depression and/or anxiety but no significant current depressive and/or anxiety symptoms, the percentage of those with daily cognitive concerns was almost twofold higher than in former players without a diagnosis or current report of depression and/or anxiety (52.2% vs. 27.3%, respectively). In former players with a diagnosis of depression and/or anxiety and current experience of moderate to severe symptoms, the percentage of those with daily cognitive problems was as high as 90.3%. This finding could be interpreted as an increased vulnerability for current daily cognitive concerns in those with a diagnosis of depression and/or anxiety, even in the absence of current symptoms (e.g., because of successful pharmacological management). In addition, compared with past players without day-to-day cognitive problems, former professional ASF players reporting daily cognitive concerns reported a lower quality of life (Hypothesis 2) and were more likely to smoke and engage in exercise at a lower level (Hypothesis 3).

If former players are, in fact, reluctant to disclose mental health symptoms but willing to report cognitive symptoms, then the assessment of cognitive difficulties might be used as a means of identifying a group of former players at risk for depression and anxiety, conditions known to reduce quality of life, for which effective treatments are available when diagnosed. Previous studies suggest that high-level football players are reluctant to seek help for mental health problems, and links have been drawn to hypermasculinity and long-time exposure to a value system reinforcing masculine concepts, such as competition and toughness.^21,31^ Given the reliance on the self-reporting of symptoms in making the diagnosis of mental health problems, this potential under-reporting of symptoms increases the risk that former players with depression and/or anxiety will be un- or misdiagnosed. If, however, former players are more likely to report cognitive symptoms, the assessment of cognitive function could be used to identify former players at higher risk for mental health problems. Although further research is needed, the combination of questions chosen for this study (Table 1) might be a valuable screening tool to engage in further conversations about cognitive challenges and conditions that former players (and individuals from similar professional groups) might be reluctant to raise with their clinicians, specifically mental health problems, even if these problems are not initially endorsed.

The close link between cognitive problems and depressive and anxiety symptoms in former professional ASF players shown in this study is in alignment with previous reports of a close relationship between the cognitive and emotional domains in other populations. Cognitive impairments are frequently reported in patients with a diagnosis of depression or anxiety.^32,33^ They have been shown to represent a marker of disease severity with the extent of cognitive problems being correlated with symptom severity and illness duration.^33,34^

The current data represent an association and do not allow for inferences about causation. We cannot determine whether among former professional ASF players, cognitive problems lead to mental health challenges, such as depressive and anxiety symptoms, whether mental health problems cause cognitive impairments, or whether third factors, such as injuries, cause both cognitive problems and mental health challenges. Previous studies, however, suggest that cognitive impairments often precede and predict emotional symptoms.^35–37^ Failure to suppress engagement with threatening stimuli (anxiety disorders), to disengage from mood-congruent thoughts or external stimuli (depression), and to apply emotion regulation strategies (both depression and anxiety disorders), all relying on adequate cognitive function, may represent key contributing factors to developing and maintaining depressive and anxiety symptoms.^38^ Moreover, alterations in the structure and function of neural networks subserving cognitive function represent a transdiagnostic core feature linked to the emotional psychopathology, providing a biological mechanism underlying the behaviorally observed link and its generalizability across mental disorders.^39,40^ Although further studies are required to illuminate the causes of the reported relationship between self-reported cognitive function and depressive and anxiety symptoms in their complex relationship with other biological, psychological, and social factors and characteristics of the players' experience, the clinical implication of gaining knowledge about mental health by assessing self-reported cognitive function represents a valuable pragmatic strategy to identify individuals in need of further mental health counseling.

Our findings must be considered in light of the study's limitations. First, we relied on self-reporting for the determination of daily cognitive problems. Therefore, we cannot confirm that such self-reports accurately reflect true cognitive deficits. Previous literature, however, suggests that self-reported cognitive problems indicate developing cognitive problems even before standard neuropsychological testing captures them.^41^ In addition, our self-report measure of cognitive function was defined through the use of a unique selection of questions from the Neuro-QOL Item Bank,^23^ which is an established reliable and validated assessment. In this population, however, it is possible that their self-perception of cognitive difficulties has been affected by widespread media reporting about later-life cognitive problems in former ASF players. Second, we included in our definition of mental health outcomes self-reports of prescribed medication for the according mental health condition or recommendations for such medications. Therefore, there were likely respondents who had these condition but were offered only non-pharmacological therapies. Because the information on symptoms and diagnoses were collected simultaneously, we cannot infer the directionality of these findings. Third, our sample represents 28.6% of eligible, potentially contactable former players. Although this response rate is similar to previous surveys of professional athlete cohorts,^42^ the possibility of a bias among the cohort cannot be excluded. Finally, additional factors, such as financial stability, socioeconomic status, self-perceived social status and support, and loss of a meaningful group role might play a role in the development and maintenance of and support-seeking for mental health challenges and might be useful to include in follow-up studies.

Our study reveals that daily cognitive problems are strongly associated with depression and anxiety among former professional ASF players. Therefore, as a primary care provider or sports medicine expert, it is important to remember that when confronted with complaints about cognitive performance, depression and/or anxiety might coexist. Validated and standardized tools are available to screen for current depressive and anxiety symptoms, including the items (including short forms for efficient assessment) of the Depression and Anxiety domains of the PROMIS^®^ (Mental Health/Emotional Distress; www.healthmeasures.net/explore-measurement-systems/promis)^27^ or the PHQ-4 (used in this study).^25^ For more formal testing, the items assessing the Fear and Sadness constructs as part of the National Institutes of Mental Health Toolbox (Emotion Battery/Negative Affect; nihtoolbox.org) should be considered.
